# Feasibility of establishing a rehabilitation programme in a Vietnamese intensive care unit

**DOI:** 10.1371/journal.pone.0247406

**Published:** 2021-03-03

**Authors:** Nguyen Thi Kim Anh, Lam Minh Yen, Nguyen Thanh Nguyen, Phung Tran Huy Nhat, Tran Thi Diem Thuy, Nguyen Thanh Phong, Pham Thi Tuyen, Nguyen Hoang Yen, Mary Chambers, Nguyen Van Hao, Thomas Rollinson, Linda Denehy, C. Louise Thwaites

**Affiliations:** 1 Hospital for Tropical Diseases, Ho Chi Minh City, Vietnam; 2 Oxford University Clinical Research Unit, Ho Chi Minh City, Vietnam; 3 Centre for Tropical Medicine and Global Health, University of Oxford, Oxford, United Kingdom; 4 University of Medicine and Pharmacy, Ho Chi Minh City, Vietnam; 5 Austin Hospital, Melbourne, Australia; 6 University of Melbourne, Melbourne, Australia; 1. IRCCS Neuromed 2. Doctors with Africa CUAMM, ITALY

## Abstract

Increasing numbers of people are surviving critical illness throughout the world, but survivorship is associated with long-term disability. In high-income settings physical rehabilitation is commonly employed to counter this and improve outcomes. These utilize highly-trained multidisciplinary teams and are unavailable and unaffordable in most low and middle income countries (LMICs). We aimed to design a sustainable intensive care unit (ICU) rehabilitation program and to evaluate its feasibility in a LMIC setting. In this project patients, care-givers and experts co-designed an innovative rehabilitation programme that can be delivered by non-expert ICU staff and family care-givers in a LMIC. We implemented this programme in adult patient with patients with tetanus at the Hospital for Tropical Diseases, Ho Chi Minh City over a 5-month period, evaluating the programme’s acceptability, enablers and barriers. A 6-phase programme was designed, supported by written and video material. The programme was piloted in total of 30 patients. Rehabilitation was commenced a median 14 (inter quartile range (IQR) 10–18) days after admission. Each patient received a median of 25.5 (IQR 22.8–34.8) rehabilitation sessions out of a median 27 (22.8–35) intended (prescribed) sessions. There were no associated adverse events. Patients and staff found rehabilitation to be beneficial, enhanced relationships between carers, patients and staff and was deemed to be a positive step towards recovery and return to work. The main barrier was staff time. The programme was feasible for patients with tetanus and viewed positively by staff and participants. Staff time was identified as the major barrier to ongoing implementation.

## Introduction

Improvements in hospital care have meant that increasing numbers of patients now survive critical illness. However, many of these patients face significant physical, cognitive and psycho-social disability after hospital discharge [1]. These impairments can be long lasting, affecting survivors’ quality of life and ability to return to normal societal roles. Both patient’s and carers’ return to work is impeded, further increasing the economic impact of critical illness [2]. Critical illness is a major concern in low and lower-middle income countries (LMICs) where the disease burden is greater and outcomes are worse than in high income settings [3]. There are increasing numbers of initiatives to improve critical care access and survivorship from critical illness in these settings and, as survival improves, it is important to ensure that long-term outcomes also improve [3,4]. However, this is challenging as in most resource-limited settings there are very limited rehabilitation services for these patients [5,6]. It is estimated that only 3% of the LMIC population requiring rehabilitation are actually receiving any, and the number of people living with disability in low income countries has doubled since 1990 [7,8].

There is level one evidence that physical rehabilitation provided in the intensive care unit (ICU) is safe and reduces physical activity limitation at hospital discharge [9]. Further evidence related to long term improvements is conflicting and hampered by the heterogeneity of patient populations studied but multidisciplinary specialist rehabilitation teams including physiotherapists and occupational therapists are costly and generally unavailable in LMIC settings like Vietnam [10,11]. In order to address problems of muscle wasting and improve functional outcome in such settings, an innovative approach to rehabilitation is required. In high-income countries, there is increasing interest in collaborative co-design and co-implementation of health services between patients, family members and medical staff. Reported benefits of this approach are that it may more specifically meet needs and is more likely to be implemented [12–14]. Whilst there is little reported literature from LMICs, further advantages in these settings include reducing requirement for specialist service providers [15,16].

In this paper, we describe a collaborative project, to design, implement and evaluate an innovative rehabilitation programme for critically ill patients with tetanus in a Vietnamese ICU. Whilst rare in high income settings, tetanus remains a significant health concern in many LMICs [17,18]. Patients with tetanus form the largest group of critically ill patients in the ICU at the Hospital for Tropical Diseases Ho Chi Minh City (HTD), staying an average 3 weeks in ICU and often several months in hospital [19]. They suffer from muscle loss, weakness and long-term disability related to this and also, importantly, a lack of improvement of muscle strength and function between ICU discharge and hospital discharge [20]. Having single musculoskeletal organ failure, these patients are uniquely representative of the impairments and activity limitations defined in post Intensive care syndrome (PICS) that result from an extended ICU and hospital stay [21,22]. Additionally, specific disease features of muscle rigidity and evoked spasm present particular challenges for rehabilitation. Whilst specially designed for patients with tetanus, we aimed that, if successful, our model could be easily adapted for use in different patient populations in other resource-limited settings. Specifically, the aims were to design a sustainable intensive care unit (ICU) rehabilitation program and to evaluate the feasibility of its delivery in a LMIC setting.

## Materials and methods

The project was carried out in the ICU and ICU recovery ward at the Hospital for Tropical Diseases, Ho Chi Minh City. This is a large tertiary referral hospital for infectious diseases serving Southern Vietnam. Patients at this hospital are treated according to a standardized protocol. Briefly, for patients with severe tetanus this includes antitoxin, sedation with benzodiazepines (intravenous reduced to oral when able to swallow). Benzodiazepines are adjusted to patients’ spasms and muscle rigidity and typically continued (although at lower dose) until hospital discharge. Severe patients require mechanical ventilation, usually with neuromuscular blocking agents (pipecuronium). The airway is secured by a primary tracheostomy which is removed usually 1 or 2 days after weaning from mechanical ventilation. Prior to this study, there was minimal specific tetanus rehabilitation–one physiotherapist was available in the ICU at doctors’ requests.

The study was approved by the Hospital for Tropical Diseases Scientific and Ethical Committee as well as the Oxford Tropical Research Ethics Committee and, as is standard at this hospital for all quality improvement and audit initiatives, the rehabilitation programme was implemented so that all patients gave written informed consent before participating. Those who did not participate received routine treatment for tetanus as described above without any specific rehabilitation programme. Subjects gave written consent for photographs and videos, including the individual in this manuscript who has given written informed consent (as outlined in PLOS consent form) to publish these case details.

### Design & development phase

We used a co-design process to devise and develop the programme that was subsequently implemented in a pilot phase with 30 patients. International experts in ICU rehabilitation spent time in the ICU and recovery ward to understand the requirements of patients and their families and to work with local ICU staff, patients and their carers. Activities involved attending ward rounds, observing nursing practices and conducting interviews and focus groups with ICU staff, patients and carers. From these interactions the requirements of the new programme were defined.

In order to minimize the number of specially-trained staff required, the programme was designed to be deliverable by non-experts and family members and to be started in ICU but continue after patients were discharged to the recovery ward. A provisional set of graded exercises was devised for patients, aiming to build key capacities and meet patient rehabilitation needs identified during the observation period–these included passive stretching to maintain muscle length and minimize joint contractures (important given tetanus symptoms), patient positioning in bed to improve lung mechanics and reduce pressure areas, functional in bed activities and sitting over the edge of the bed to improve strength, and standing and mobilization. To evaluate contextual feasibility and acceptability of individual exercises, these exercises were piloted in a limited number of patients over a week-long period by nurses and family care-givers after training by the international rehabilitation experts. At the end of the week a focus-group with ICU staff was held to facilitate feedback. The specific exercises that could be correctly delivered and were acceptable to staff, patients and carers were then incorporated into the final programme.

The final programme consisted of a stepwise progression consisting of six phases. Each phase consisted of a series of between 5 and 11 different exercises, each of which were prescribed in 20 minute sessions (whilst in ICU) or sets of repetitions (in recovery ward). After completion of one phase (with transitions defined according to pre-defined targets–see S1 Table), the patient progressed through subsequent phases. Exercises progressed from passive movement to assisted and active movements encompassing strength, function and mobility. A full description of this programme is given in S1 Table. Transition from passive to active phases was deemed possible when patients were able to carry out 3 of 5 criteria that assess if a patient was awake and co-operative as defined by De Jonghe [23]. Pre-defined safety rules for stopping a rehabilitation session were also defined [24]. In order to support programme delivery, printed leaflets and videos were produced showing the exercises and how to progress these after improvement. The exact format of these was finalized in a participatory workshop with patients and family care-givers.

### Implementation & evaluation phase

The programme was implemented at the Hospital for Tropical Diseases for a 5-month period and feasibility evaluated according to number and completeness of exercise sessions performed, adverse events, acceptability and barriers to the programme from patients, carers and staff perspectives. A period of 5 months was chosen for the implementation phase, aiming to recruit between 20 and 30 patients. This sample size was expected to allow saturation in qualitative outcomes and also detect an estimated 80% completion as prescribed with 95% confidence.

Rehabilitation team members attended daily ICU ward rounds and screened for eligible patients. Eligible patients were those patients ≥18 years-old with severe tetanus who were with a carer able to speak Vietnamese and before hospital admission were able to walk unaided. Patients aged > 70 years old were excluded from the study. Eligible patients were identified during morning ward rounds and once the attending doctor confirmed that that passive movement did not provoke spasm, rehabilitation could be started (See S1 Table). Rehabilitation providers were experienced ICU doctors and nurses with extensive experience of care of tetanus patients. Training by international rehabilitation experts included three specific face-to-face courses of 1 week in duration, informal supervision and observation delivering the final program. Family carers were able to assist in sessions and were trained and supervised by the local rehabilitation providers. Rehabilitation sessions were delivered by the rehabilitation providers twice daily on week-days. Generally, sessions were not given on weekends due to lack of staff. Sessions were approximately 30 minutes’ duration. In the ICU phases, rehabilitation was primarily provided by trained providers but as patients moved to the recovery ward, carers participated more in delivering the rehabilitation.

Evaluation was carried out concurrently with implementation. All prescribed rehabilitation sessions were recorded, including exercises completed and any adverse events. Reasons for patients not completing the session or particular exercises were also recorded. Patient and staff perspectives were obtained using questionnaires and semi-structured interviews conducted by a trained senior nurse with in a combined session where a face-to-face short questionnaire was followed by interview (S1 File). These were performedinthe 28 surviving patients prior to hospital discharge. Responses were recorded via a 5-point Likert scale and through free text (S1 File). For staff perspectives were derived from questionnaires were given to 30 ICU nurses following focus group discussions which included all nurses on shifts at these times (S1 File). All questionnaires and interviews were conducted according to guidelines. Results were analysed using quantitative and qualitative methodologies. For descriptive statistics, median (interquartile range) are given for skewed continuous data and number (%) for categorical data. For qualitative data, all responses were analyzed for thematic content, coded into broad categories by two of the study team. As this is a feasibility study with a single cohort, no comparisons have been. All statistics were analysed in R Version 4.0.03 (R Corp Vienna, Austria).

## Results

### Feasibility evaluation

A total of 30 patients were enrolled into the programme between 10^th^ February and 3^rd^ July 2019. Baseline and demographic features of these patients are given in Table 1.

**Table 1:** Participant characteristics.

Variable	Median (IQR)
Age (years)	53 (46–57)
Male sex n (%)	24 (80%)
BMI (kg/m^2^)	22 (18.7–23.5)
Time from first symptom to admission (days)	3 (2–4)
Incubation period (days)	6 (5–7)
Period of onset (hours)	36 (24–72)
ICU length of stay (days)	22 (18–24)
Hospital length of stay (days)	30 (26–33)
Mechanical ventilation n (%)	28 (93%)
Duration mechanical ventilation (days)	17 (13–21)
Autonomic nervous system dysfunction n (%)	12 (40%)
Total diazepam during hospital stay (mg)	143 (65–240)
Total midazolam during hospital stay(mg)	2200 (1850–2575)
Total pipecuronium during hospital stay (mg)	480 (220–660)
Median days receiving pipecuronium	11 (9–16)

Rehabilitation was commenced a median (inter quartile range (IQR)) 14 (10–18) days after admission. All patients were receiving benzodiazepine sedation and had a tracheostomy in situ at programme commencement. Eight patients were still receiving neuromuscular blocking agents (pipecuronium) and 27/30 (90%) of patients were receiving mechanical ventilation at the time of commencement. Each patient received a median (IQR) of 26 (23–39) rehabilitation sessions, equating to a median (IQR) 98 (96–100) percent of intended (prescribed) sessions. Sessions were delivered twice daily to patients on almost all scheduled days once the programme had commenced, with prescribed sessions delivered over a median (IQR) of 14 (10–18) days. A median of 1 (0–1) sessions per patient were not delivered. The commonest reason for this was removal of their tracheostomy tube during the time scheduled for the session (12/30 40% patients). Other reasons included fatigue (4/30 13% patients), fever (2/30 6% patients) or being transferred to another ward at the scheduled time.

Of delivered sessions, almost all patients were able to complete all components of the session (97%-99% complete of phases 1,2,4–6). The exception from this was phase 3 when only 84% of delivered sessions were complete. This was almost exclusively due to patients not able to complete assisted sitting on the edge of the bed in ICU. The median duration of each phase was between 2 and 4 days. Time from ICU admission to first sitting was a median of 22 (17–25) days; or median 6 (4–9) days from starting rehabilitation whereas time to the assisted standing was a median 23 (19–25) days from admission, or 7 (5–12) days from starting rehabilitation.

No serious adverse events were related to the rehabilitation programme. Two patients died due to complications of tetanus (septic shock and myocardial infarction) and these events were not related to rehabilitation.

### Patients and staff perspectives

All patients wanted to participate in rehabilitation, with 27/28 (96%) expressing they ‘very much’ wanted to do so. No patient had previously met a physiotherapist or rehabilitation therapist, but when asked about preferences for guiding the exercises, 21/28 (68%) of respondents were happy with any of doctor, nurse or specialist-rehabilitation technician providing rehabilitation, whereas 9% preferred only doctors or doctors and physiotherapists to provide this. All participants reported positive experiences, identifying that it was easy to carry out, useful and they believed led to a faster recovery. Challenges identified included fatigue, reliance on help from relatives and fear of falling when carrying out the exercises without professional providers. Nevertheless, 23/25 (92%) of patients felt they would be able to safely carry out the exercises at home with a relative’s help if necessary, whereas two participants felt they were still too weak to do this. All felt that the information booklet was very helpful (Table 2).

**Table 2:** Results from patients/relatives and healthcare nurses about rehabilitation program during hospitalization.

	Patients/Family	Nurses
**Acceptibility**	• Easily able to understand and follow all exercises • No extreme tiredness to prevent the patients’ practice	• Easily able to understand and guide all exercises for patients • Happy to involve relatives in rehabilitation phases
**Barriers**	• Joint stiffness/pain • Fatigue • Need assisstance from relatives • Fear of falling	• Have little time for both patient’s care and additional rehabilitation exercises • No specialist rehabilitation doctor or trained physical therapists to support sessions • Obese patients may present a difficulty
**Benefits**	• A positive step to take oneself towards returning to normal life • Quick recovery after ICU discharge–particularly towards returning to work • Social interaction with the therapists	Enhanced the relationship between healthcare staff and patients/family
**Resource**	Printed materials/Videos are helpful with the explicit instruction on exercises	

In questionnaires given to nurses, nurses had a median 11 (IQR 4–13) years’ experience in nursing. All nurses reported that they felt the programme was useful to the patient. Reasons stated were that it was perceived to prevent muscle wasting, reduced rigidity, improved mobility and allowed faster recovery. 24/29 (83%) of the nurses felt that the supporting booklet was ‘very’ or ‘extremely’ helpful. All ICU nurses felt that they would be able to deliver almost all ICU phases of the programme when they had finished their other caring and administrative duties. However, time was the most frequently identified barrier to implementing the programme (21/28, 75% respondents) followed by sedation or delirium preventing co-operation (13/28, 46%) and patients’ pain (8/28, 29%).

## Discussion

In order to reduce the global burden of disability, WHO has identified the need for early rehabilitative interventions, but acknowledges the challenges of providing these in LMICs given the lack of resources and sufficiently trained providers [7]. Our study aimed to evaluate the feasibility of an early ICU-based exercise rehabilitation programme to address a proven need in patients with tetanus. Prior to this, there were no formal rehabilitation programmes in our hospital, although patients with tetanus had been identified as a group experiencing long-term functional disability following hospital discharge [20].

Our primary aim was to create a feasible and sustainable programme suitable for a resource-restricted setting [25]. Our results show that staff, patients and carers perceived the programme to be useful and beneficial. Providers were able to deliver the exercises as intended almost all of the time and patients were able to move steadily through the progression as they improved. Rules for transition between phases appeared to be appropriate, with participants spending a similar number of sessions in all phases. No serious adverse events occurred and, importantly, ICU staff felt that they were able to deliver the programme to their patients. Only one exercise (sitting on the edge of the bed whilst the participant remained in ICU) was challenging to implement. Despite this the median day to commence sitting over the edge of the bed after starting rehabilitation was 6 days. This is similar to reports from studies in high income ICUs [26]. The patient population studied in Vietnam had only single organ failure and perhaps this contributed to this finding being similar even though the staff had less time. Lack of staff or time were the reasons most commonly recorded for not doing this, however it may be that the lack of experience and confidence was also a reason staff felt unable to perform this since it requires skills in moving and supporting patients safely while still encouraging exercises. Other studies from high income ICUs also report similar lack of time issues. The supportive literature and videos produced were felt to be helpful and easy to understand. For staff with little prior training in exercise, this was an important component of the success of the implementation [27,28].

We believe a vital element of the success of our progamme was the co-design process we employed throughout this project. This approach is increasingly used and advocated in high-income settings, including ICU [13,29,30]. In our project, experts, staff, patients and families worked together to design and implement the rehabilitation programme. This allowed participant and providers views to be incorporated throughout the design process, and particularly in the case of supportive literature, these were often quite different from experts prior expectations. We utilized task shifting/sharing to allow non-expert providers to deliver the programme. Similar methods have been used successfully to deliver other healthcare interventions in both well-resourced and resource-restricted settings [31–34]. Generally, in our study, task shifting was well received–particularly by family carers, but we note that in other clinical healthcare contexts it has also been associated with negative outcomes such as threatened professional identity and devaluation of tasks [33,35].

Our project specifically aimed to evaluate an ICU based rehabilitation programme with respect to both short-term delivery feasibility and long-term implementation. We believe the exercise programme designed was safe, suitable for our cohort of patients and could be delivered by non-expert providers. Although designed for use in tetanus, our programme could easily be adapted for other critically ill patients. Nevertheless, we encountered several barriers to longer-term implementation and sustainability. Whilst all participants and staff felt that the rehabilitation programme was valuable and beneficial, most of the delivery in practice was performed by a few key individuals with help from patients’ relatives. The advantage of this is that it allowed continuity between sessions and a uniform approach to implementation, however it meant that many staff were still not confident to deliver the programme at the end of the period. In questionnaire responses, staff commonly reported that rehabilitation was a task to be carried out after ‘normal work’, suggesting that it was viewed as distinct from normal patient care and an additional load for staff. Nurse: patient ratios in our ICU are similar to many LMICs, with one nurse caring for 3 or 4 patients, consequently ‘normal work’ duties are likely to occupy the majority of a nurse’s time, particularly when patients have many other nursing requirements. Further engagement work is therefore needed if a larger group of staff are to be retained as providers in the future.

We did not attempt to evaluate efficacy of the programme. However, to improve sustainability, improving ICU doctors’ and nurses’ knowledge of rehabilitation and its place in multidisciplinary care is essential and, as part of this, demonstrating the efficacy of physical rehabilitation is vital. Staff who perceive there is a benefit for their patients are more likely to participate and encourage other team members to do so. A future intervention study should also include health economic analysis, particularly taking account of indirect costs. In this report we have not attempted to compare outcomes from other ICU rehabilitation studies as we do not feel this is meaningful given the study population.

## Conclusion

A graded, progressive exercise-based rehabilitation programme was safe and feasible to implement in a LMIC ICU. Further work is, however, necessary to ensure sustainability of this programme, particularly engaging ICU staff and ultimately demonstrating its efficacy. Currently, evidence in favour of ICU rehabilitation is based predominately on studies in high income settings but it is crucial to show the efficacy of physical rehabilitation interventions in LMIC settings where patient case-mix and potential interventions may be very different.

## Supporting information

S1 Fig Carer/Patient supportive material for ward and home based rehabilitation. (DOCX)

S1 Table Elements of the exercise programme. Note there are no chairs available in the ICU and very few in the recovery ward).
(DOCX)

S1 File Participant and staff questionnaire and interview guide Vietnamese (original) and English translation. (PDF)
