# Supplementary figures and images for: Feasibility of establishing a rehabilitation programme in a Vietnamese intensive care unit
Source: PLoS One. 2021 Mar 3;16(3):e0247406. doi: 10.1371/journal.pone.0247406 (PMC7928504; doi:10.1371/journal.pone.0247406)

### **S2 Fig. Carer/Patient supportive material for ward and home based rehabilitation.**


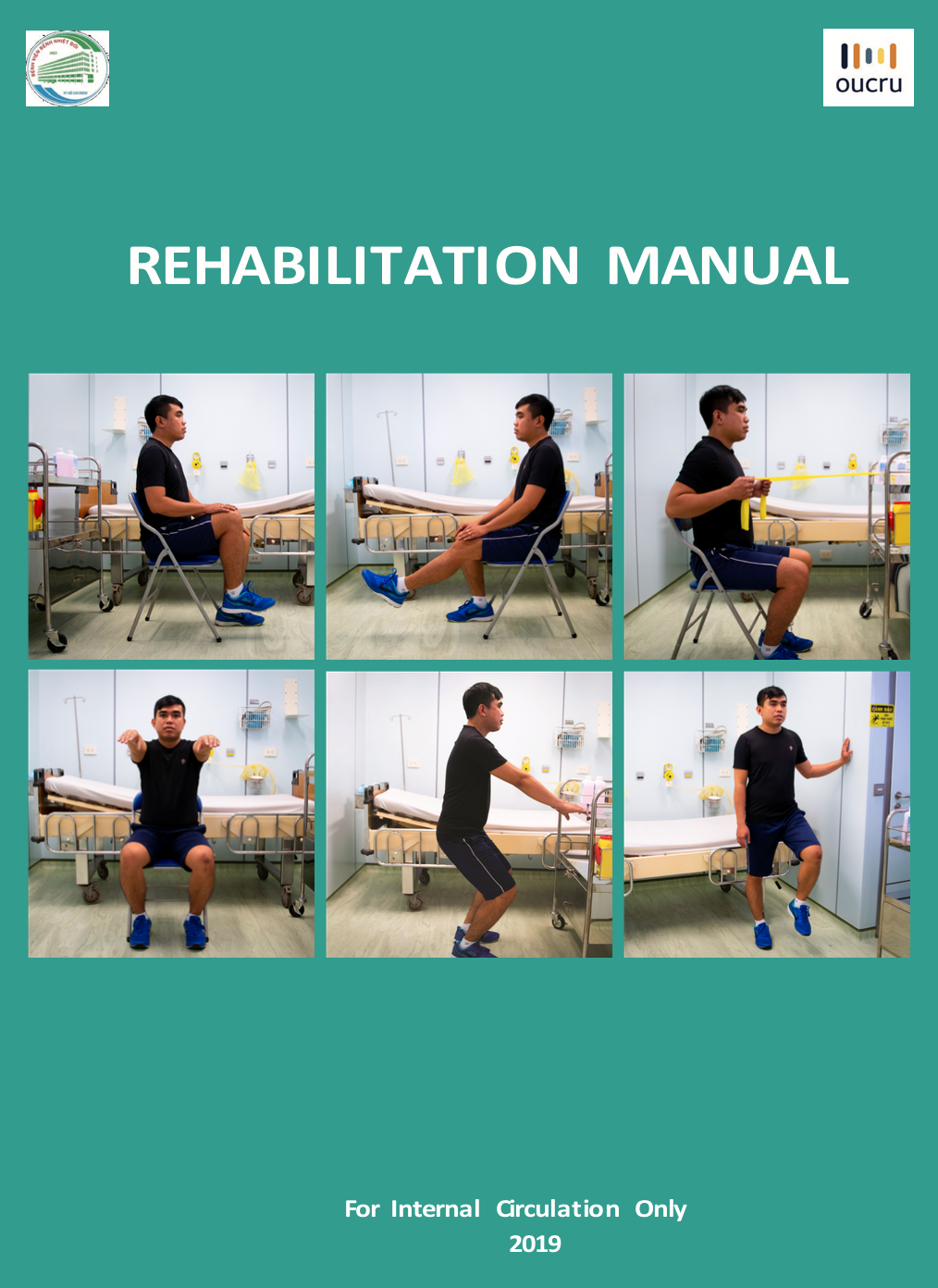

Supplement: S1 Fig — (DOCX) [file pone.0247406.s001.docx]
