# Supplementary material for: Feasibility of establishing a rehabilitation programme in a Vietnamese intensive care unit
Source: PLoS One. 2021 Mar 3;16(3):e0247406. doi: 10.1371/journal.pone.0247406 (PMC7928504; doi:10.1371/journal.pone.0247406)
Supplement: S1 Table — Note there are no chairs available in the ICU and very few in the recovery ward). (DOCX) [file pone.0247406.s002.docx]

## **S1 Table: Elements of the exercise programme.** (Note there are no chairs available in the ICU and very few in the recovery ward)

| Phase | Criteria for starting | Exercises | Dose |
| --- | --- | --- | --- |
| Phase 1: Passive movement in ICU | Passive movement not provoking spasms | Head up position 30⁰  Knee flexion/extension  Hip flexion/ abduction  Foot dorsiflexion/ plantarflexion  Shoulder abduction/ flexion  Fingers/ hand positioning | Passive movements; 3 movements each stretch for 20 minutes 2 x day |
| Phase 2: Assisted movement to unassisted movement in ICU | Responding to 3 out of 5 verbal commands  Able to assist rolling in bed | Head up position 30⁰  Knee flexion/extension  Hip flexion/ abduction  Foot dorsiflexion/ plantarflexion  Bilateral arm raising  Bridging in bed  Sitting on edge of the bed | 3-5 repetitions of each exercise -in total 20 minutes 2 x day:  Sitting on edge of the bed: once per day |
| Phase 3: Assisted movement in sitting position in ICU | Able to sit on the edge of the bed | Knee flexion/ extension  Foot dorsiflexion/ plantar flexion  Bilateral arm raising:  Forward and lateral reach (balance)  Assisted standing | 5 repetitions of each exercise -in total 20 minutes 2 x day:  Assisted standing: once per day |
| Phase 4: Recovery ward seated exercises | Able to sit on bed or walk out of room | Bridging in bed  Seated marching on the spot  Seated knee extension  Seated shoulder flexion  Seated shoulder row  Seated  foot and ankle pumps / calf raises. | 1 sets of 8 repetitions, increasing to 3 sets of repetitions with theraband resistance.  2 sessions a day:  Assisted walking 3 x day (10-20m) |
| Phase 5: Exercises in corridor | Able to walk out of room and completed 3 sets of 8 repetitions of exercises in Phase 4. | Walking out of room – holding rail  ¼ squat  Marching on the spot  Standing heal raise  1 leg stand  Weighted shoulder flexion/ abduction  Weighted shoulder shrug  Seated shoulder row  Walking in the corridor | Initially 2 set of 8 repetitions, 3 sets of 8 repetitions with theraband resistance.  2 sessions a day  Walking assistance only for safety: 3 times daily (20-50m) |
| Phase 6: Exercises in corridor | Completion of 3 sets of phase 5 exercises | Sit to stand  Standing on one leg  Unsupported double heal raise  Wall push ups  Walking | Initially 2 set of 8 repetitions, 3 sets of 8 repetitions with theraband resistance.  2 sessions a day  Walking unaided: 3 times daily ( as far as possible.50m |
