# Supplementary material for: Feasibility of establishing a rehabilitation programme in a Vietnamese intensive care unit
Source: PLoS One. 2021 Mar 3;16(3):e0247406. doi: 10.1371/journal.pone.0247406 (PMC7928504; doi:10.1371/journal.pone.0247406)
Supplement: S1 File — (PDF) [file pone.0247406.s003.pdf]

BẢNG CÂU HỎI CHO BỆNH NHÂN/THÂN NHÂN

CÂU HỎI	LOẠI CÂU TRẢ LỜI
	0 hoàn toàn không – 5 hoàn toàn có
Q1. Các bài tập có khó hiểu/khó thực hiện ?	0 1 2 3 4 5
	0 hoàn toàn không – 5 rất nhiều
Q2. Các bài tập có làm bạn mệt ?	0 1 2 3 4 5
Q3. Bạn có muốn tập PHCN/giúp BN tập ?	0 1 2 3 4 5
Q4. Tài liệu in có hữu ích đối với bạn ? Thêm hay bớt ? Tại sao ?	0 1 2 3 4 5
Q5. Bạn gặp khó khăn gì khi tập PHCN ?	
Q6. Bạn thấy chương trình PHCN thế nào ? (Ấn tượng)	
Q7. Bạn thích tập với ai ?	BS/ĐD/KTV VLTL
Q8. Khi bạn có thắc mắc, bạn có được giải quyết ngay ?	Có/Không
Q9. Bạn đã từng gặp KTV VLTL trước đây chưa ?	Có/Không
Q10. Lợi ích nhất và bất tiện nhất khi bạn tập PHCN ?	
Q11. Khó khăn khi tập PHCN ở nhà ?	
Q12. Thân nhân nghĩ gì khi bạn tập PHCN ?	
Q13. Yếu tố gì chi phối khi bạn tập PHCN tại nhà ?	
Q14. ĐD/KTV hướng dẫn có khó hiểu ?	Có/Không
Q15. Bạn có cảm thấy an toàn nếu tập PHCN ở nhà ? Tại sao ? Chúng tôi giúp gì cho bạn được ?	Có/Không – cụ thể
Q16. Yếu tố chính nào giúp bạn tập chủ động được ?	
Q17. Yếu tố chính nào ngăn cản bạn không tập chủ động được ?	
Q18. Kinh nghiệm của bạn khi tập PHCN tại bệnh viện ?	

Chú thích: BN: bệnh nhân; BS: bác sĩ; KTV: kĩ thuật viên; PHCN: phục hồi chức năng; VLTL: vật lý trị liệu

BẢNG CÂU HỎI DÀNH CHO NHÂN VIÊN Y TẾ

CÂU HỎI	LOẠI
	0 hoàn toàn không – 5 hoàn toàn có
Q1. Các bài tập có khó hướng dẫn cho BN?	0 1 2 3 4 5
Q2. Bài tập nào bạn có thể làm tốt nhất ?	
Q3. Bài tập nào có thể chỉnh sửa để tốt hơn ?	
Q4. Booklet hay video theo bạn là tốt ?	Video/ booklet/cả hai
	0 hoàn toàn không – 5 rất nhiều
Q5. Bạn có muốn TN giúp BN tập PHCN?	0 1 2 3 4 5
Q6. Tài liệu in có hữu ích đối với bạn?	0 1 2 3 4 5
Q7. Tài liệu video có hữu đối với bạn?	0 1 2 3 4 5
Q8. Booklet hay video có cần chỉnh sửa gì không ?	
Q9. Bạn có gặp khó khăn gì khi tập PHCN cho BN?	
Q10. Bạn có đề nghị gì giúp việc tập PHCN được tốt hơn?	
Q11. Bạn có kinh nghiệm gì khi tập PHCN cho BN ?	
Q12. Suy nghĩ của bạn về chương trình PHCN ?	
Q13. Bạn có khó khăn khi cần trao đổi với ĐD/BS Khoa khi tập cho BN ?	
Bạn làm ĐD bao nhiêu năm ? Bằng cấp ?	

Chú thích: BN: bệnh nhân; BS: bác sĩ; ĐD: điều dưỡng; PHCN: phục hồi chức năng; TN: thân nhân

PATIENT QUESTIONNAIRE

QUESTION	TYPE OF ANSWER
Q1. Were the exercises difficult to understand or to do?	0 not at all – 5 impossible
Q2. Was you tired when doing the exercises?	0 not at all – 5 very much so
Q3. Do you want to undertake/help patient undertaking exercises?	0 not at all – 5 very much so
Q4. Did you find the printed book helpful?	0 not at all – 5 very much so
Q5. What were the challenges with undertaking exercise program?	Open question
Q6. Can you tell me what your impression of the exercise program was?	Open question
Q7. Who you want to guide you to undertake exercises?	Rehab technician, doctor or nurse
Q8. Did you find the program physio and nurse consultations were available at the right time ?	Yes/ no
Q9. Have you ever meet a physiotherapist/rehab technician?	Yes/no
Q10. What was the most/least valuable aspect of participating in this study?	Open question
Q11. What are your thoughts on the challenges with undertaking exercise program at HOME?	Open question
Q12. What are the thoughts of your family / friends on you undertaking the exercise program?	Open question
Q13. What factors are relevant to undertake excercise program at home ?	Open question
Q14. Did you feel supported and able to be guided with your exercises by the physiotherapist ?	Yes/ no
Q15. Do you think it is safe to exercise at HOME? Why? How could we have helped?	Yes/ no specify
Q16. What are the main factors that enable you to be active at the moment ?	Open question

Q17. What are the main factors that prevent you from being physically active?	Open question
Q18. What is your experience regarding to the exercise program during hospitalization?	Open question

NURSE QUESTIONNAIRE

QUESTION	TYPE
Q1. Do you find the exercises difficult to guide for the patient?	0 not at all – 5 very much so
Q2. Which is the best useful exercise could you guide?	Open question
Q3. Which exercise of the program do you feel could have been improved? Do you have any suggestions for how these aspects could have been improved?	Open question
Q4. Do you find the booklet or video helpful?	Video/ booklet/ both
Q5. Do you want relatives to help the patient?	0 not at all – 5 very much so
Q6. Is the booklet useful?	0 not at all – 5 very much so
Q7. Is the video useful?	0 not at all – 5 very much so
Q8. What aspects of the booklet or video do you feel could have been improved?	
Q9. Can you tell me about any challenges that you encountered in delivering the exercise?	Open question
Q10. Do you have any suggestions for how these aspects could have been improved?	Open question
Q11. Tell me about your experiences with the rehabilitation program	Open question
Q12. What was your overall impression of the intervention?	Open question
Q13.Did you have any difficulty in interacting with doctors or nurses when carrying out the program	Open question

Working time (year)
Degree
